# Comparative analysis of clinical efficacy of unilateral biportal endoscopic and open transforaminal lumbar interbody fusion in the treatment of lumbar degenerative

**DOI:** 10.3389/fsurg.2025.1487168

**Published:** 2025-01-23

**Authors:** Tao Ma, Xiaoshuang Tu, Junyang Li, Yongcun Geng, Jingwei Wu, Senlin Chen, Dengming Yan, Ming Jiang, Gongming Gao, Luming Nong

**Affiliations:** ^1^ Department of Orthopedics, Nanjing Medical University, Jiangsu, China; ^2^ Department of Orthopedics, Dalian Medical University, Liaoning, China; ^3^ Department of Orthopedics, The Affiliated Changzhou No.2 People's Hospital with Nanjing Medical University, Changzhou, China

**Keywords:** degenerative lumbar disease, lumbar fusion, minimally invasive, UBE, ULIF

## Abstract

**Objective:**

To study the clinical efficacy of unilateral biportal endoscopic lumbar interbody fusion (ULIF) and transforaminal lumbar interbody fusion (TLIF) in the treatment of lumbar degenerative diseases, and to compare perioperative indicators, radiological outcomes, and paraspinal muscle –atrophy resulting from these two different surgical methods.

**Background:**

Transforaminal lumbar interbody fusion (TLIF) is widely acknowledged as an efficacious surgical modality for alleviating low back pain. In recent years, unilateral biportal endoscopic lumbar interbody fusion (ULIF) has gained increasing application.

**Methods:**

We recorded the basic information of patients who underwent single-segment ULIF or TLIF for the first time in our hospital from May 2021 to November 2022, including age, gender, BMI, diagnosis, and surgical segment. Perioperative indicators such as estimated blood loss, operation time, postoperative hospital stay, and complications were observed in both groups. Clinical efficacy was assessed preoperatively and at 1 month, 3 months, and 12 months postoperatively using the Visual Analogue Scale (VAS) and the Oswestry Disability Index (ODI). Patient satisfaction was evaluated using the modified Macnab criteria. The displacement of the fusion device was also assessed. x-rays were taken preoperatively, at 3 months postoperatively, and at 12 months postoperatively to observe fusion device displacement and measure the intervertebral disc height of the upper and lower segments. The Cobb angle was used to measure lumbar lordosis and segmental lumbar lordosis. CT scans at 3 months postoperatively were used to observe intervertebral fusion, including bridging trabeculae, endplate cysts, and screw loosening. MRI at 1 year postoperatively was used to manually trace the cross-sectional area of the paraspinal muscles to compare muscle atrophy.

**Results:**

A total of 150 patients were included in the study, with 71 patients in the ULIF group and 79 patients in the TLIF group. No statistically significant disparities were observed between the two groups with respect to age, gender, BMI, diagnosis, and surgical segment**.** The estimated blood loss in the ULIF group was 108.78 ± 58.3 ml, which was significantly less than that in the TLIF group at 199.44 ± 84.91 ml (*p* < 0.001). The postoperative hospital stay was shorter in the ULIF group (*p* = 0.020), although the operation time was longer for ULIF. There were no significant differences in complications between the two groups. Patients in the ULIF group experienced quicker relief from back pain postoperatively, but there were no significant differences between the ULIF and TLIF groups in the VAS, ODI, and satisfaction rates at the final follow-up. At 3 months postoperatively, the ULIF group demonstrated a higher incidence of bridging trabeculae, a lower incidence of endplate cysts, and less fusion device displacement**.** There were no significant differences between the two groups in the correction of segmental lumbar lordosis (SL) and overall lumbar lordosis (LL). Additionally, the ULIF group showed less muscle damage.

**Conclusion:**

ULIF has the advantages of reducing pain in the short term, less blood loss, and shorter hospital stays. Its more precise handling of the intervertebral space reduces the occurrence of endplate cysts and fusion device displacement, which has certain significance in preventing delayed fusion and nonunion. However, ULIF requires a longer operation time, which increases potential risks for elderly patients or those with poor nutritional status. Although ULIF causes less damage to the bony structure, it has not shown a significant advantage in improving adjacent segment degeneration.

## Background

Low back pain constitutes one of the most prevalent reasons prompting patients to seek medical attention. Studies have shown that more than 60% of cases involving lower back pain will recur within a year, and 15%–40% of individuals with newly onset lower back and leg pain will experience chronic pain or recurrent episodes (1). Currently, there are no prospective randomized controlled trials to determine non-surgical treatments, but when stubborn symptoms or associated sensory-motor impairments do not respond to conservative treatments, surgical intervention becomes crucial (2). Lumbar fusion surgery represents an effective modality for mitigating the symptoms of degenerative lumbar conditions**.** It includes several different surgical approaches, such as anterior lumbar interbody fusion (ALIF), posterior lumbar interbody fusion (PLIF), and transforaminal lumbar interbody fusion (TLIF). The surgeon determines the surgical approach based on the patient's symptoms, signs, auxiliary examinations, and their personal expertise. Compared to anterior lumbar interbody fusion, TLIF and posterior lumbar interbody fusion are more widely used and have similar clinical outcomes in improving symptoms of lumbosacral pain. The TLIF technique can be considered an improvement over posterior lumbar interbody fusion, utilizing a unilateral transforaminal approach to the disc space, partially removing facet joints to expose nerves laterally, thereby reducing nerve traction and the risk of iatrogenic nerve injury. In addition, TLIF causes less damage compared to posterior lumbar interbody fusion by reducing the need for spinous process removal, thereby preserving the integrity of the posterior column. Meta-analyses have confirmed that TLIF offers advantages in terms of reduced blood loss and shorter surgical times. Multiple studies have demonstrated that TLIF results in good clinical outcomes for patients postoperatively (3). TLIF does not require exposure of the contralateral intervertebral foramen for fusion, significantly reducing the risk of nerve injury. Although TLIF surgery achieves extensive decompression of neural structures and stabilizes the operative segment, as a traditional open surgery, its main drawbacks include larger surgical trauma and disadvantages for early patient mobility compared to minimally invasive techniques. Additionally, it may cause damage to bony structures and alter biomechanical properties post-fusion, thereby increasing the risk of adjacent segment degeneration (4). The management of the intervertebral space is crucial for interbody fusion. Bridging bone trabeculae are important indicators for evaluating the fusion process, while the appearance of vertebral endplate cysts is considered an effective predictor of poor fusion. In recent years, endoscopic techniques have gradually matured, offering advantages such as preserving normal tissue structures, minimal trauma, fewer complications, and fast postoperative recovery. Recently, the unilateral biportal endoscopic fusion technique (ULIF) has gained widespread application (2). ULIF not only shares a comparable operative scope with TLIF but also features endoscopic and working channels that facilitate direct access to the intervertebral space for endplate preparation, thereby enabling a more direct observation of the extent of endplate manipulation. This approach minimizes the likelihood of excessive residual nucleus pulposus or damage to the bony endplates. Furthermore, the enhanced precision of decompression achieved through endoscopy can mitigate damage to bony structures. The procedural steps of ULIF are similar to those of TLIF. Therefore, the objectives of this study are: firstly, to compare the postoperative clinical efficacy of ULIF and TLIF; secondly, to observe whether ULIF's advantages in intervertebral space handling lead to better fusion results; and thirdly, to determine whether ULIF causes significantly different muscle damage compared to TLIF.

## Methods

This study was approved by the local Ethics Committee (approval number 2023YLJSA012). Patient data were collected from May 2021 to November 2022 for ULIF and TLIF treatments conducted by our surgical team. The inclusion criteria for patients in the study are as follows: (1) undergoing initial single-segment ULIF or TLIF surgery; (2) diagnosed with degenerative lumbar conditions including spondylolisthesis, segmental instability, or degenerative disc disease with ineffective conservative treatment for more than 3 months; (3) exhibiting symptoms, signs, and auxiliary examinations congruent with the diagnosis; (4) capable of cooperating in responding to pertinent inquiries. Exclusion criteria include: (1) revision surgery; (2) severe spinal scoliosis; (3) presence of vertebral fractures or tumors; (4) spinal infectious diseases. Criteria for choosing ULIF instead of TLIF are as follows: (1) Minimize the surgical wound. (2) Reduce perioperative pain; (3) the optimization of surgical area and lighting equipment during the operation (4) rapid return to society after surgery.

**Figure 1:**
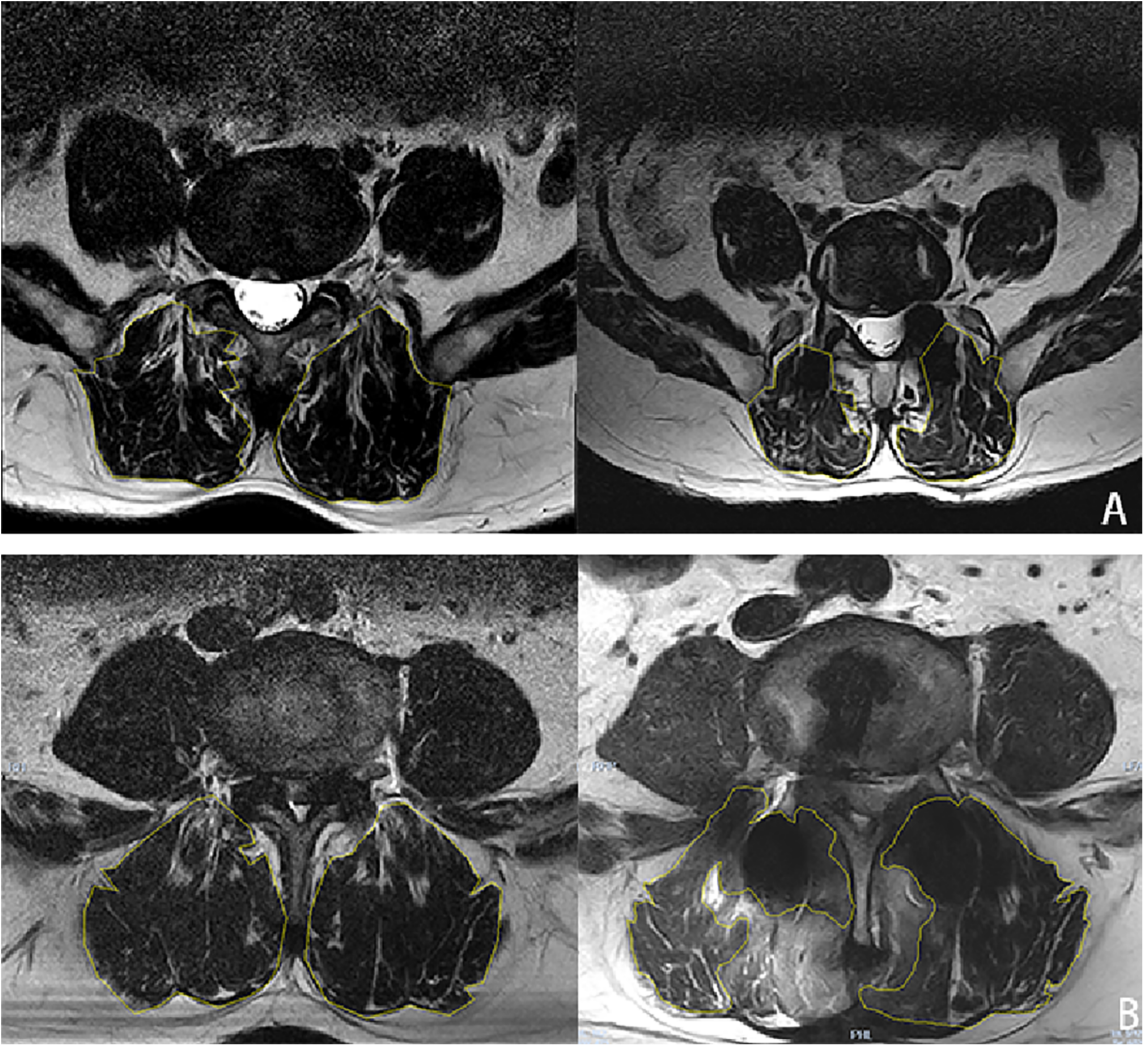
Cross-sectional area of paraspinal muscle was hand-drawn using image J image processing software, and **(A)** is cross-sectional measurement of paraspinal muscle 1 year after ULIF in a 62-year-old male. **(B)** Shows cross-sectional area measurement of paraspinal muscle in a 65-year-old man at 1 year after TLIF.

Document perioperative parameters for patients, encompassing surgical duration, postoperative complications, and surgical blood loss quantified via Nadler's and Gross's methodologies. Nadler's formula calculates blood volume as follows: Blood Volume = k1 × Height (m) + k2 × Weight (kg) + k3, where for males, k1 = 0.3669, k2 = 0.03219, k3 = 0.6041; and for females, k1 = 0.3561, k2 = 0.03308, k3 = 0.1833. Gross's formula calculates total blood loss as: Total Blood Loss = Blood Volume × (Hct pre-op + Hct post-op)/(2 × Average Hct), where Average Hct = (Hct pre-op + Hct post-op)/2. Postoperative clinical outcomes of patients were assessed using Visual Analog Scale (VAS) and Oswestry Disability Index (ODI) at preoperative, 1-month, 3-month, and 1-year intervals. Patient satisfaction was evaluated using the modified Macnab criteria. x-rays were taken preoperatively, at 3 months postoperatively, and at 12 months postoperatively to measure the height of upper and lower segment intervertebral spaces. Intervertebral space height measurement was calculated as (height of anterior intervertebral space + height of posterior intervertebral space)/2 (5). Using Cobb angle measurements to assess lumbar lordosis and segmental lumbar lordosis, where lumbar lordosis measures the angle from the L1 upper endplate to the S1 upper endplate on x-ray, and segmental lumbar lordosis measures the angle between the upper edge of the superior vertebral body and the lower edge of the inferior vertebral body of the operative intervertebral space. Postoperatively at three months, CT scans are used to observe vertebral fusion, including bridging bone trabeculae, vertebral endplate cysts, and screw loosening. Vertebral endplate cysts are defined as new cysts >2 mm appearing at any level of the operated segment. One year postoperatively, MRI was used to observe paraspinal muscle atrophy, and Image J software was used to manually trace and measure the cross-sectional area of the paraspinal muscles (Figure 1).

### Procedure

**Figure 2:**
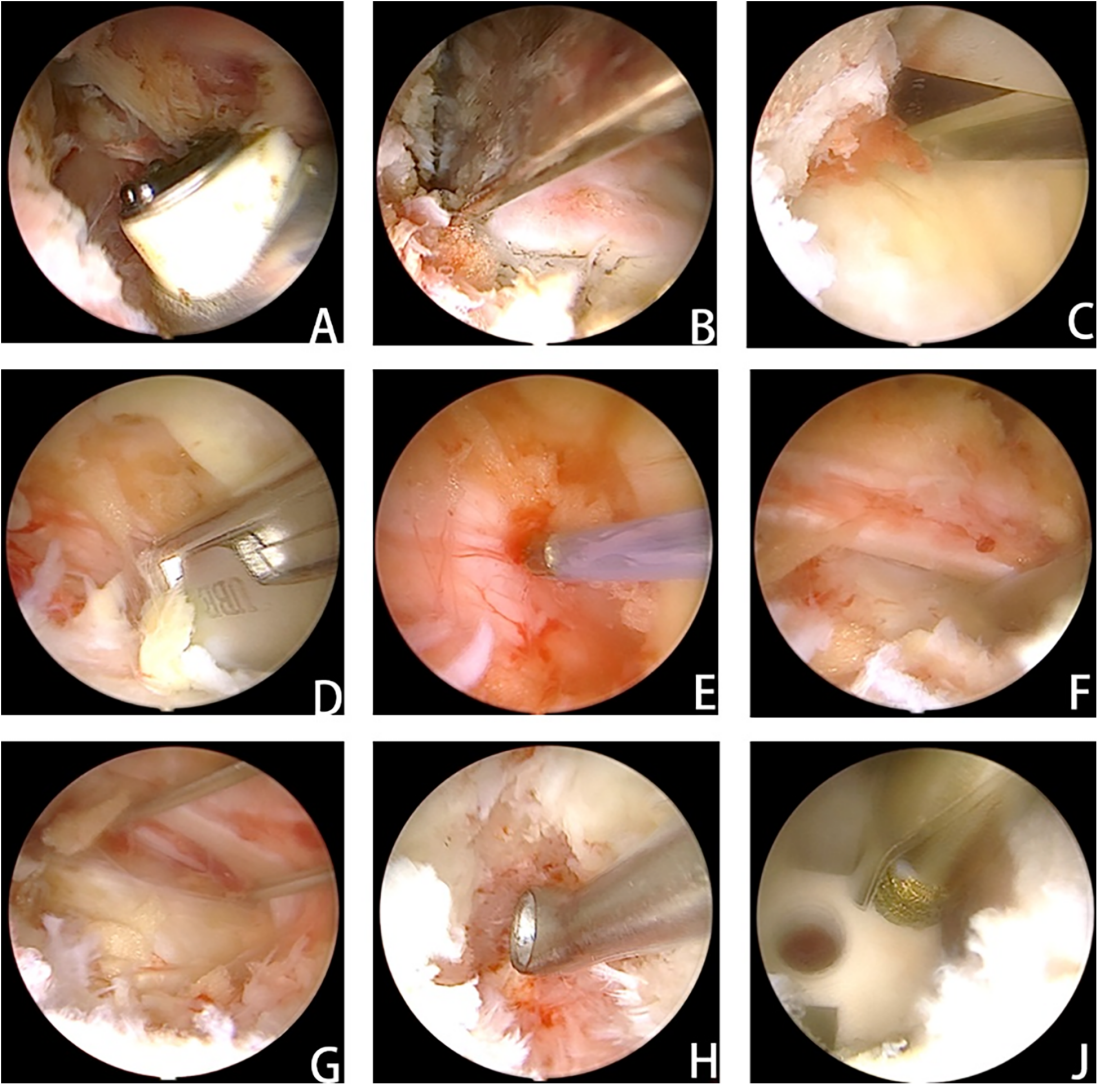
After the cartilage endplate was carefully and completely removed, the bone fragments collected in advance were placed in front of the responsible intervertebral space after testing the model. After tamping, the fusion cage was inserted. **(A)** Clearance of soft tissue to establish access and adequate exposure of bony structures. **(B)** Resection of the inferior articular process. **(C)** Partial laminectomy. **(D)** Removal of the ligamentum flavum. **(E)** Adequate exposure of the dural sac and prompt hemostasis. **(F)** Nerve exploration and release. **(G)** Kirschner wires were used to retract the nerve and expose the visual field of the intervertebral space. **(H)** The intervertebral space was processed under the visual field of the intervertebral space. **(J)** The cage was placed, and the depth of cage insertion was evaluated with a grinding drill for adjustment.

ULIF Procedure: The patient is placed prone under general anesthesia. Using a C-arm fluoroscope, the operative level is positioned vertically relative to the ground, and frontal fluoroscopy is used to confirm and mark the channel establishment site. Standard disinfection and draping are performed, and waterproof draping is applied. Initiating from the midline of the intervertebral space, symmetric vertical incisions are meticulously made approximately 0.5–1.0 cm lateral to the outer margin of the vertebral arches. The working channels are about 1 cm wide, and observation channels are approximately 0.5 cm wide to accommodate the endoscope. Various ULIF instruments are inserted through the working channels for the procedure (Figure 2). Both channels are bluntly dissected through soft tissues to minimize direct muscle damage. Continuous saline irrigation is used to improve surgical visibility; inadequate irrigation can affect visibility. Under endoscopic guidance, tissues are exposed down to bony structures, with timely hemostasis using electrocautery throughout. Depending on the specifics, drills, bone knives, and chisels are used under endoscopy for precise partial vertebral plate and facet joint removal, with autologous bone collected for grafting. The ligamentum flavum is excised to expose the dura mater or nerve roots for further decompression. The large operational space of ULIF allows for lateral recess and contralateral decompression. Neurolysis probes are used to explore and release nerves, with preemptive hemostasis. RF probes are used to excise intervertebral discs, and under clear endoscopic vision, the endplates are prepared by removing residual nucleus pulposus until visible blood vessels are seen. A funnel-shaped cannula is used for autologous bone grafting into the intervertebral space, followed by insertion of a polyetheretherketone interbody fusion device under fluoroscopic observation. Finally, all instruments are removed, and conventional percutaneous bilateral pedicle screw fixation is performed. A drainage tube is placed as well.

TLIF: After successful induction of general anesthesia, the patient is positioned prone. A midline incision approximately 8 centimeters long is made in the lower back, centered on the operative intervertebral space. The incision penetrates through the skin, subcutaneous tissue, and deep fascia. Starting from the more symptomatic approach, the procedure involves fully exposing the vertebral body, superior and inferior facet joints, and the vertebral notch of the upper endplate. Subsequent procedures are similar to ULIF.

### Statistical analysis methods

Statistical analysis was conducted using SPSS for Windows (version 26.0; SPSS Inc.). The continuous variables that conformed to the normal distribution were presented as the mean ± standard deviation (x ± s). The independent sample *t*-test was adopted for the comparison between two groups of measurement data that conformed to the normal distribution, while the paired sample *t*-test was employed for the comparison within the same indicator group. The repeated measures analysis of variance was utilized for the comparisons at multiple time points. The categorical variables were represented by frequencies or percentages and analyzed using the chi-square test. A *P* value < 0.05 was regarded as statistically significant.

## Results

### Study population

A total of 150 patients were included in this study, and all surgeries were performed by the same experienced surgical team. There were 71 participants in the ULIF group and 79 in the TLIF group, and none were lost to follow-up. There was no significant difference in baseline data between the two groups, including age, gender, BMI, fusion segment, and diagnosis (Table 1).

**Table 1:** Baseline data for ULIF and TLIF.

	ULIF (*n* = 71)	TLIF (*n* = 79	*P* value
Sex (male/female)	33/38	25/54	0.063
Age (years)	58.69 ± 15.7	62.49 ± 11.09	0.129
BMI(kg/m^2^)	25.53 ± 4.42	26.88 ± 5.91	0.131
Fusion levels			0.941
L3/4	6	7	
L4/5	39	41	
L5/S1	26	31	
Diagnosis			0.962
lumbar spondylolisthesis	42	45	
Segmental instability	19	22	
lumbar spondylolysis	10	12	

### Perioperative indicators

The blood loss of the ULIF group was 108.78 ± 58.3 ml, which was significantly lower than that of the TLIF group (199.44 ± 84.91 ml), and the difference was statistically significant (*p* < 0.001). This is a consequence of the smaller trauma caused by ULIF. The shorter postoperative hospital stay (*p* = 0.020) was only 6.1 ± 1.71 days in the ULIF group compared with 7.17 ± 1.88 days in the TLIF group, and the ULIF operation time was longer (175.82 ± 75.19 min), while the TLIF group was only 111.75 ± 38.08 min, and there was a significant difference in the length of hospital stay and operation between the two groups (*p* < 0.001) (Table 2). Patients in the ULIF group can be discharged from the hospital earlier and return to daily life as soon as possible, which is important for older patients, and we believe that this is the result of less damage to bone and soft tissues and good decompression, but the need for patients to experience longer anesthesia increases the potential risk. There was one case (1.4%) of dural sac tears in the ULIF group and one case (1.3%) of dural sac tears in the TLIF group, both of which were treated conservatively, and there was no statistically significant difference in complications between the two groups (*p* > 0.05).

**Table 2:** Perioperative period index.

	ULIF	TLIF	*P* value
Operation time (h)	175.82 ± 75.19	111.75 ± 38.08	<0.001*
Blood loss (ml)	108.78 ± 58.30	199.44 ± 84.91	<0.001*
Postoperative hospital stay (d)	6.10 ± 1.71	7.17 ± 1.88	0.020*

* Significant difference between two groups.

### Clinical efficacy

The symptoms of the two groups were significantly relieved in the last follow-up, and the VAS and ODI scores were significantly improved compared with those before surgery. At the postoperative follow-up to 1 month, the improvement of VAS low back pain score and ODI score in the ULIF group was significantly better than that in the TILF group, which were 2.36 ± 0.83, 29.99 ± 7.08 and 2.7 ± 0.94 and 33.93 ± 8, respectively, with a statistically significant difference (*p* = 0.019 and *p* = 0.001). This meant that ULIF had a greater advantage in improving short-term pain, but there was no statistically significant difference between the two groups during subsequent follow-up periods (*P* > 0.05). At the last follow-up, the VAS low back pain score decreased from 5.68 ± 1.11 to 1.27 ± 0.77, the VAS leg pain score decreased from 4.73 ± 1.64 to 1 ± 0.85, and the ODI score decreased from 63.27 ± 9.29 to 7.08 ± 6.1. In the TLIF group, the VAS low back pain score decreased from 5.71 ± 1.26 to 1.24 ± 0.79, the VAS leg pain score decreased from 4.82 ± 1.44 to 0.99 ± 0.9, and the ODI score decreased from 63.36 ± 9.68 to 8.09 ± 5.15. There was no statistically significant difference in VAS leg pain scores between the two groups at each follow-up time (Table 3). According to the modified Macnab criteria, there was no significant difference in 65 cases of excellent, 5 cases of good, 1 case of acceptable and 0 cases of poor (excellent rate) in the ULIF group and 70 cases of excellent, 7 cases of good, 1 case of acceptable and 1 case poor (excellent rate of 97.5%) in the ULIF group (*p* > 0.05).

**Table 3:** Comparisons of VAS back, VAS leg, and ODI scores between ULIF and TLIF groups.

	ULIF (*n* = 71)	TLIF (*n* = 79)	*P* value
VAS back			
Preoperation	5.68 ± 1.11	5.71 ± 1.26	0.866
1 month after operation	2.36 ± 0.83	2.70 ± 0.94	0.019*
3 months after operation	1.52 ± 0.67	1.55 ± 0.96	0.790
12 months after operation	1.27 ± 0.77	1.24 ± 0.79	0.832
VAS leg			
Preoperation	4.73 ± 1.64	4.82 ± 1.44	0.720
1 month after operation	2.64 ± 0.96	2.44 ± 1.01	0.206
3 months after operation	1.61 ± 0.73	1.49 ± 0.86	0.393
12 months after operation	1.00 ± 0.85	0.99 ± 0.9	0.852
ODI			
Preoperation	63.27 ± 9.29	63.36 ± 9.68	0.954
1 month after operation	29.99 ± 7.08	33.93 ± 8.00	0.001*
3 months after operation	14.62 ± 5.59	15.90 ± 4.83	0.105
12 months after operation	7.08 ± 6.1	8.09 ± 5.15	0.243

VAS, visual analogue scale; ODI, oswestry disability index.

* Significant difference between two groups.

### Radiological findings

At the 3-month postoperative interval, there were 10 vertebral endplate cysts, 1 case of screw loosening, 1 case of fusion device displacement, and 65 cases of bridging trabecular bone in the ULIF group. In the TLIF group, there were 28 vertebral endplate cysts, 1 case of screw loosening, 7 cases of fusion device displacement, and 60 cases of bridging trabecular bone at 3 months after operation. Compared with the TLIF group, the ULIF group had more trabecular bridging bones, fewer vertebral endplate cysts and fusion device displacement, and the difference was statistically significant (*p* 0.010, 0.003, and 0.031, respectively) (Table 4). We believe that this result is consistent with our hypothesis, considering that it is a positive result due to the advantages of ULIF in the treatment of vertebral spaces. The paravertebral muscle area of the ULIF group and the TLIF group was 1,829.5 ± 125.5mm^2^ and 1,828.9 ± 152.7mm^2^, respectively, were measured by MRI, and there was no significant difference (*p* > 0.05). One year after operation, the paravertebral muscle area of the two groups was measured and the paravertebral muscle atrophy of the TLIF group was 1,724.4 ± 144.0 mm^2^, which was statistically significant compared with the ULIF group (1,820.6 ± 141.7 mm^2^) (*p* < 0.001) (Table 4). This result suggests that ULIF is less damaging to the paravertebral muscles than TLIF.

**Table 4:** Radiographic findings in the Two groups.

	ULIF (*n* = 71)	OTLIF (*n* = 79)	*P* value
VEC	10 (14.1%)	28 (35.4%)	0.003
PSL	1 (1.4%)	1 (1.3%)	0.724
CM	1 (1.4%)	7 (8.8%)	0.031
CTB	65 (91.5%)	60 (75.9%)	0.010
Preoperation paravertebral muscle area	1,829.5 ± 125.5 mm^2^	1,828.9 ± 152.7 mm^2^	0.981
12 months after operation paravertebral muscle area	1,820.6 ± 141.7 mm^2^	1,724.4 ± 144.0 mm^2^	<0.001
	*P* = 0.070	*P* < 0.001	

VEC, vertebral endplate cyst; PSL,e wqCTB, continuous trabecular bone.

**Figure 3:**
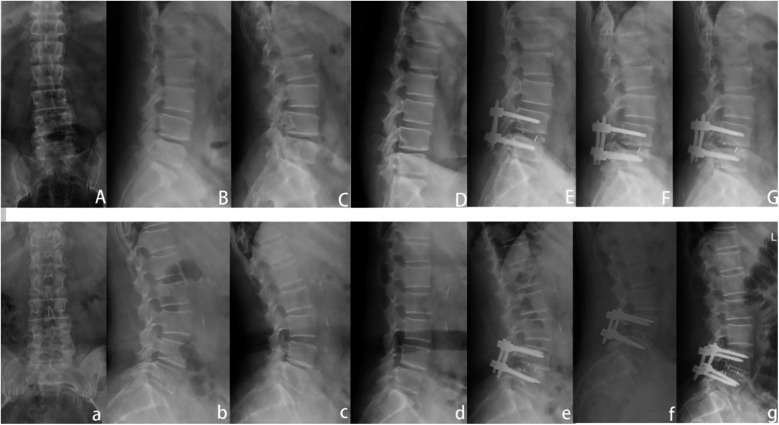
Presentation of typical cases of the two groups of patients. **(A**–**G)** A 55-year-old male patient in the ULIF group. They were lumbar anteroposterior, lateral, hyperextension, hyperflexion, 1 month postoperative lateral, 3 months postoperative lateral, and 1 year postoperative lateral. **(a**–**g)** a 62-year-old woman in the TLIF group.

One year after surgery, the intervertebral space of adjacent segments decreased to varying degrees in both groups. In L3/4, the L2/3 intervertebral space height was 10.76 ± 1.25 mm, the L4/5 intervertebral space height was 10.56 ± 0.86, the L2/3 intervertebral space height was 10.48 ± 0.87, and the L4/5 intervertebral space height was 9.97 ± 0.44 in the TLIF group, and there was no statistical difference between the two groups (*p* > 0.05). In L4/5, the L3/4 intervertebral space height was 9.61 ± 2.6, the L5/S1 intervertebral space height was 9.56 ± 2.48, the L3/4 intervertebral space height was 9.69 ± 1.99, and the L5/S1 intervertebral space height was 9.71 ± 1.74 in the L5/S1 group, and the L4/5 intervertebral space height was 10.01 ± 1.55 in the ULIF group and 9.75 ± in the TLIF group1.36, and there was no statistical significance between the two groups (*P* > 0.05) (Table 5). One year after operation, x-rays showed that the segmental lordosis angle in the ULIF group was 11.37 ± 1.74, the lumbar lordosis angle was 39.82 ± 0.99, the segmental lordosis angle in the TLIF group was 11.91 ± 2.1, and the lumbar lordosis angle was 40.72 ± 0.99 in the L4/5 segment, and the segmental lordosis angle was 11.94 ± 1.89, the lumbar lordosis angle was 39.28 ± 4.18, and the segmental lordosis angle in the TLIF group was 12.86 ± 3.42, the lumbar lordosis angle was 39.88 ± 3.75, and in the L5/S1 segment, the segmental lordosis angle was 11.91 ± 2.35, the lumbar lordosis angle was 39.17 ± 5.02, and the TLIF group, the segmental lordosis angle was 11.57 ± 1.53, and the lumbar lordosis angle was 39.84 ± 4.63. There was no significant difference in the correction of lumbar lordosis angle and segmental lumbar lordosis angle between the ULIF group and the TLIF group (*p* > 0.05) (Table 5). Typical cases of patients in both groups are shown (Figure 3).

**Table 5:** Results of x-ray and angle measurements one year after surgery.

	L3/4			L4/5			L5/S1		
	ULIF	TLIF	*p*	ULIF	TLIF	*p*	ULIF	TLIF	*p*
Preoperative segmental lordosis Angle	7.78 ± 0.84	8.63 ± 0.91	0.163	8.99 ± 1.86	8.86 ± 2.01	0.765	9.94 ± 1.69	9.69 ± 1.77	0.583
Postoperative segmental lordosis	11.37 ± 1.74	11.91 ± 2.1	0.670	11.94 ± 1.89	12.86 ± 3.42	0.136	11.91 ± 2.35	11.57 ± 1.53	0.531
Preoperative lumbar lordosis Angle	38.49 ± 0.76	38.51 ± 1.99	0.979	38.18 ± 4.10	38.79 ± 4.12	0.507	38.32 ± 5.26	38.82 ± 4.95	0.712
Postoperative segmental lordosis	39.82 ± 0.99	40.72 ± 0.99	0.186	39.28 ± 4.18	39.88 ± 3.75	0.501	39.17 ± 5.02	39.84 ± 4.63	0.605
The height of the intervertebral space of the upper segment before operation	11.12 ± 1.08	11.16 ± 0.91	0.949	11.12 ± 2.33	11.28 ± 2.24	0.754	11.04 ± 1.90	11.05 ± 1.48	0.981
The height of the upper segment intervertebral space after operation	10.76 ± 1.25	10.48 ± 0.87	0.692	9.61 ± 2.60	9.69 ± 1.99	0.880	10.01 ± 1.55	9.75 ± 1.36	0.513
Preoperative lower segment intervertebral space height	11.58 ± 0.93	11.38 ± 0.89	0.736	10.01 ± 2.61	10.13 ± 1.98	0.819	–	–	
The height of intervertebral space of lower segment after operation	10.56 ± 0.86	9.97 ± 0.44	0.213	9.56 ± 2.48	9.71 ± 1.74	0.749			

## Discussion

For patients with lumbar instability, fusion of the affected segment is necessary. In this study, the ULIF group demonstrated early pain relief, less intraoperative blood loss, faster recovery, and shorter hospital stays compared to the TLIF group. Early mobilization helped patients return to normal life quickly. However, at the last follow-up, both groups showed similar clinical outcomes without statistical differences. ULIF surgery prolonged operative time and posed increased surgical risks for elderly patients undergoing prolonged prone anesthesia. With technological advancements, minimally invasive surgery has been widely promoted, supported by multiple studies showing comparable clinical efficacy. A meta-analysis indicated that endoscopic lumbar fusion surgery shows favorable short-term outcomes, with significant improvements in VAS back pain score, VAS leg pain score, and ODI score (6, 7). This result is consistent with our research findings, indicating that endoscopic lumbar fusion surgery has significant advantages in improving short-term clinical outcomes. Some scholars have achieved good results using single-channel endoscopic fusion, but it has limitations such as restricted visibility and limited operating space, for example, inadequate lateral recess decompression, thereby narrowing its indications (8). ULIF technology overcomes some of the limitations of single-channel endoscopy and offers an operating space similar to TLIF surgery. Therefore, we consider ULIF to be a promising surgical approach. However, some argue that ULIF increases the invasiveness compared to single-channel methods due to the addition of an extra channel. Hence, we also quantitatively studied the damage to muscle tissue. One of the objectives of lumbar spine fusion surgery is the achievement of intervertebral bone fusion. Therefore, preventing postoperative non-union is crucial for the patient's prognosis.

Severe displacement of the fusion cage can directly precipitate fusion failure or induce symptoms attributable to compression of the dural sac or nerve roots. Displacement of the fusion cage may arise from several factors: initially, an excessive residual nucleus pulposus within the intervertebral space can envelop the fusion cage, impeding bony trabecular ingrowth; subsequently, inadequate endplate preparation impacts the contact area between the fusion cage and the upper and lower endplates; furthermore, excessive excision of bony structures, such as partial facet joint removal during surgery, undermines the stability of the posterior column; finally, inappropriate selection or placement of the fusion cage material, type, or bone graft material can result in compression or micromotion.

In our study, both groups of patients used fusion cages made of the same materials. The difference lies in TLIF relying more on the surgeon's experience for intervertebral space handling, which increases the risk of inadequate endplate preparation or damage to the bony endplates. During ULIF surgery, the dual-channel approach allows direct access into the intervertebral space, providing clear visualization under the microscope of the endplate preparation. The anatomical relationship between the cartilaginous and bony endplates is fully exposed, facilitating precise removal of residual nucleus pulposus and observation of blood sinus formation, ensuring optimal contact area between the bone graft and fusion area. Therefore, we believe ULIF can better facilitate intervertebral fusion, reducing the incidence of fusion cage displacement. Consequently, we posit that ULIF can more effectively facilitate intervertebral fusion, thereby diminishing the incidence of fusion cage displacement. Moreover, the lower prevalence of vertebral endplate cysts in the ULIF group further substantiates its efficacy. The development of vertebral endplate cysts may be attributable to micro-movements between the endplate and the fusion cage, potentially influenced by the materials employed in the fusion cage (9).

SutSumimoto et al.'s study suggests that anatomical factors directly contribute to paraspinal muscle injury. Increasing strength in the lower back is crucial for maintaining lumbar stability. Therefore, minimizing muscle atrophy during surgery is of paramount importance. ULIF achieves decompression and fusion under endoscopic guidance through two channels, employing blunt dissection to minimize muscle damage. Entry into the multifidus muscle interspace reduces muscular trauma to traction injury without substantive destruction. This approach effectively protects paraspinal muscles, aiding in early postoperative pain relief and long-term chronic pain reduction, thereby enhancing postoperative quality of life and facilitating early return to daily activities for muscle conditioning. In our study, ULIF patients demonstrated significant improvement in rapid recovery from preoperative lower back pain. Preoperatively educated patients exhibited varied changes in paraspinal muscle after one year, with ULIF patients showing significantly larger cross-sectional areas and less fat infiltration on MRI compared to TLIF patients. These findings indicate that, under these multifaceted considerations, ULIF minimizes paraspinal muscle damage and better alleviates symptoms for patients. Lumbar spine fusion stabilizes the affected segments, restoring sagittal balance of the lumbar spine.

ULIF and TLIF have many similarities. Their surgical approaches and the anatomical structures they encounter are similar. Moreover, the surgical instruments used in TLIF can also be utilized in ULIF. Therefore, during ULIF surgery, even if unexpected situations arise, the two longitudinal incisions can be connected in a timely manner, converting it to an open surgery. The difference is that compared to TLIF, ULIF maintains a clear surgical field under the endoscope with timely hemostasis using a high-frequency electric knife and continuous irrigation with saline solution, preventing bleeding from affecting the surgical view. This avoids inadequate decompression and iatrogenic injuries caused by a compromised surgical field. In this study, none of the patients experienced incomplete decompression. The unilateral biportal endoscopic technique allowed for meticulous endplate preparation, minimizing the risk of endplate damage and providing more favorable conditions for postoperative interbody fusion.

Finally, our study has several limitations. First, the study was not randomized; second, the sample size was not large enough, and the follow-up period was not long enough; third, the manual measurement of angles and areas may have errors and cannot completely eliminate the interference of metal artifacts after lumbar fusion.

## Conclusion

In summary, we believe that ULIF is a minimally invasive lumbar fusion surgery that is safe and effective for treating degenerative lumbar diseases. It offers similar clinical outcomes to TLIF but with the advantages of being less invasive, causing less bleeding, and promoting quicker recovery. While maintaining the operative range, ULIF also reduces damage to the paraspinal muscles. The more precise handling of the intervertebral space in ULIF decreases the occurrence of vertebral endplate cysts and cage displacement, which is significant in preventing delayed fusion and non-union. However, ULIF requires longer surgical time, which poses potential risks for elderly patients or those with poor nutritional status. Although ULIF is less invasive, the improvement in low back and leg pain symptoms is similar to that of TLIF. Despite less destruction of bony structures with ULIF, it does not show a significant advantage in improving adjacent segment degeneration.

This article has been approved by the Ethics Committee of Changzhou Second Hospital.

## Data Availability

The original contributions presented in the study are included in the article/Supplementary Material, further inquiries can be directed to the corresponding authors.
